# Highly sensitised individuals present a distinct Treg signature compared to unsensitised individuals on haemodialysis

**DOI:** 10.3389/frtra.2023.1165320

**Published:** 2023-10-30

**Authors:** C. Dudreuilh, S. Basu, O. Shaw, H. Burton, N. Mamode, F. Harris, T. Tree, P. Nedyalko, M. Terranova-Barberio, G. Lombardi, C. Scottà, A. Dorling

**Affiliations:** ^1^ Department of Inflammation Biology, School of Immunology and Microbial Sciences, King’s College London, London, United Kingdom; ^2^ Centre for Nephrology, Urology and Transplantation, School of Immunology and Microbial Sciences, King’s College London, London, United Kingdom; ^3^ Synnovis Clinical Transplantation Laboratory, Guy’s Hospital, London, United Kingdom; ^4^ Peter Gorer Department of Immunobiology, King’s College London, London, United Kingdom; ^5^ NIHR Guy’s and St Thomas’ Biomedical Research Centre at Guy’s and St Thomas NHS Foundation Trust, St Thomas’ Hospital, London, United Kingdom

**Keywords:** highly sensitised, regulatory T cells, haemodialysis, T follicular regulatory cells, sensitisation

## Abstract

**Introduction:**

Highly sensitised (HS) patients represent up to 30% of patients on the kidney transplant waiting list. When they are transplanted, they have a high risk of acute/chronic rejection and long-term allograft loss. Regulatory T cells (Tregs) (CD4^+^CD25^hi^CD127_lo_) are *T* cells involved in the suppression of immune alloresponses. A particular subset, called T follicular regulatory T cells (Tfr, CXCR5^+^Bcl-6^+^), is involved in regulating interactions between T effectors and B cells within the germinal centre and can be found in peripheral blood. Therefore, we wanted to identify specific subsets of Tregs in the peripheral blood of HS individuals.

**Methods:**

We recruited prospectively healthy volunteers (HV) (*n* = 9), non-sensitised patients on haemodialysis (HD) (*n* = 9) and HS individuals, all of whom were on haemodialysis (*n* = 15).

**Results:**

We compared the Treg phenotypes of HV, HD and HS. HS patients had more CD161^+^ Tregs (*p* = 0.02) and more CD45RA^−^CCR7^−^ T effectors (Teffs) (*p* = 0.04, memory Teffs able to home to the germinal centre) compared to HVs. HS patients had more Bcl-6^+^ Tregs (*p* < 0.05), fewer Th1-like Tregs, more Th2-like Tregs (*p* < 0.001) and more CD161^+^ (*p* < 0.05) Tregs compared to HD patients. This population has been described to be highly suppressive. HD had a deficiency in a Th17-like CD161^+^ effector Treg cluster (cluster iii., CCR6^+^CCR4^+^CXCR3^−^ CD39^+^CD15s^+^ICOS^−^CCR7^−^CD161^+^) (*p* < 0.05).

**Discussion:**

This is the first study presenting a deep Treg phenotype in HS patients. We confirmed that HS patients had more of a Th17-like CD161^+^ effector Treg from population III (CD4^+^CD25^hi^CD127_lo_CD45RA^−^) compared to non-sensitised patients on HD. The clinical relevance of this highly suppressive Tregs population remains to be determined in the context of transplantation.

## Introduction

Highly sensitised (HS) patients represent up to 30% of patients on the kidney transplant waiting list. The new UK allocation scheme should be beneficial in prioritising HS patients for a transplant, but partly due to the COVID-19 pandemic and the lack of high-risk transplants being performed, it has not been possible to clearly identify its full impact to date. However, if we extrapolate from what happened in the US with a similar allocation scheme (1), the new UK allocation system may not benefit the most highly sensitised individuals (calculated reaction frequency (crF) 95%–100%), leading to their accumulation on the transplant waiting list.

Finding matched transplants for HS patients is challenging; even with a living donor they usually have to undergo pre-transplant optimisation, including desensitisation, delisting or participating in the UK sharing scheme. When transplanted, even with a well-matched donor, they have a higher risk of both acute and chronic rejection and long-term allograft loss (2). Regulatory T cells (Tregs, CD4^+^CD25^hi^CD127_lo_) are *T* cells involved in the suppression of immune alloresponses. A particular subset, called T follicular regulatory *T* cells (Tfr, CXCR5^+^Bcl-6^+^), is involved in regulating T effectors and *B* cell interactions within the germinal centre and can be found in the peripheral blood (3). Other Treg subsets T-bet^+^ CD45RO^−^ and T-bet^−^ CD45RO+ Tregs have been associated with the presence of donor-specific antibodies (DSA) and DSA+ antibody-mediated rejection (ABMR), respectively (4–6). The aim of the present study was to assess if we could identify specific subsets of Tregs in highly sensitised individuals compared to healthy volunteers (HVs) and individuals on haemodialysis.

## Materials and methods

### Patients and samples

This study used samples from three cohorts of individuals. Two cohorts were patient recruits from studies initiated and conducted at King’s College London, whereas the third used leucocyte cones from NHS Blood and Transplant (NHSBT). Informed consent was obtained from all participants after the nature and possible consequences of the studies were explained. The first group included sensitised patients (cRF > 50%) enrolled into the “Antibody Incompatible Transplantation - a prospective study” (AIT–IRAS project ID 204733; reference no. 16/WM/0370, West Midlands, Coventry & Warwickshire ethics committee, Research Ethics Committee 22/02/2019). All patients recruited by the first author (CD) between 2019 and 2021 (*n* = 14) were included. Samples were taken at enrolment and at 3-monthly periods thereafter (maximum number 4). Participants to this study provided written informed consent before inclusion. The second group of patients included haemodialysis controls included from the “Sensitisation develop in Kidney Patients (SIKP) study” (IRA project ID 276643; reference no. 21/SC/0156). Ethical approval was obtained from the South Central–Hampshire B Research Ethics Committee. Patients enrolled in the trial were identified as dialysis patients who had not been sensitised (cRF = 0) and were enrolled by the first author (CD). HV controls were recruited using leucocyte “cones” obtained through a leukoreduction system from anonymised healthy donor peripheral blood obtained from the National Blood Service (NHSBT, Tooting, London, UK) with informed consent and ethical approval (Institutional Review Board of Guy’s Hospital; reference no. 09/H0707/86/).

### Isolation of peripheral blood mononuclear cells

All the samples were obtained from local haemodialysis units for SIKP and AIT and from NHSBT for leucocyte cones. Samples were processed using a local SOP by a single operator (CD). The peripheral blood mononuclear cells (PBMCs) were isolated using Lymphoprep separation (Lymphoprep; Stem Cell, Canada) and used fresh [resuspended in AIM V® media (Life Technologies, UK)] with 10% human AB serum (Sigma-Aldrich)–AIMV/AB.

### Phenotyping panel

PBMCs were incubated with Near IR LIVE/DEAD (Invitrogen, USA) for 15 min in a cell incubator, at 37°C, containing 5% CO_2_. After washing, cells were stained with titrated amounts of fluorochrome-conjugated monoclonal-Ab. The Tregs panel consisted of: ICOS (C398.4A), CXCR5 (J252D4, CD25 (BC96), CCR7 (G043H7), CD3 (UCTH1), CCR6 (G034E3), CD69 (FN50), PD-1 (EG12.2H7), Helios (22F6), Ki67 (Ki67) and CD45RO (UCHL-1), all from BioLegend (San Diego, CA, USA); CXCR3 (1C6/CXCR3), CD15s (CSLEX1), CCR4 (1G10), CD4 (SK3), CD45RA (Hi100), CD39 (TU66), Bcl-6 (K112–91), GATA-3 (L50-B23) and CTLA4 (BNI3), all from BD Biosciences (Franklin Lakes, NJ, USA); CD127 (EBioRDR5), FOXP3 (PCH101) and T-bet (4B10), all from ThermoFisher (Waltham, MA, USA); and CD161 (191B8, Miltenyi, Bergisch Gladbach, Germany). The list of all the antibodies, clones, colours and quantities are available in Supplementary Materials Tables S1 and S2. Cells were stained in FACS buffer [as per Stroukov et al. (7)] for 15 min in a cell incubator, at 37°C, containing 5% CO_2_. After incubation, cells were washed, fixed [1% paraformaldehyde with 99% phosphate-buffered saline (PBS)], then washed with PBS 5% FCS and stored at 4°C before acquisition within 24 h on a BD LSRFortessa flow cytometer (KCL BRC Flow Cytometry Laboratory), using Diva software (BD, Ashland, OR, USA). Samples were then imported in FlowJo (BD) and Cytobank (Beckman Coulter) for unsupervised analyses; CITRUS was run using equal numbers of Tregs (CD4^+^CD25^hi^CD127_lo_) or Teffs (CD4^+^ non-Tregs), or Tregs events per sample and the following clustering channels: CXCR5, ICOS, CCR7, CXCR3, CD161, CCR6, CD15s, CCR4, CD69, PD-1, CD45RA and CD39 for extracellular markers; and FOXP3, Helios, GATA3, Bcl-6, T-bet, Ki67, CTLA4 and CD45RO for intracellular markers. Samples with a cell viability <60% were not used in the analysis.

### Statistical analysis

The statistical analyses were performed using GraphPad Prism. *P*-values < 0.05 were considered statistically significant. Wilcoxon matched-pairs signed-rank tests were used for non-parametric paired data; Mann–Whitney tests were used for non-parametric unpaired data.

**Figure 5:**
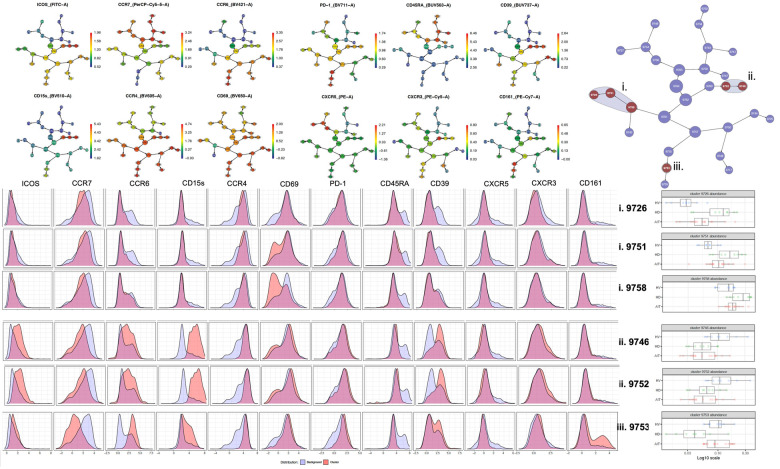
CITRUS analysis of Tregs from extracellular panel comparing HV, unsensitised HD and AIT patients.

**Figure 7:**
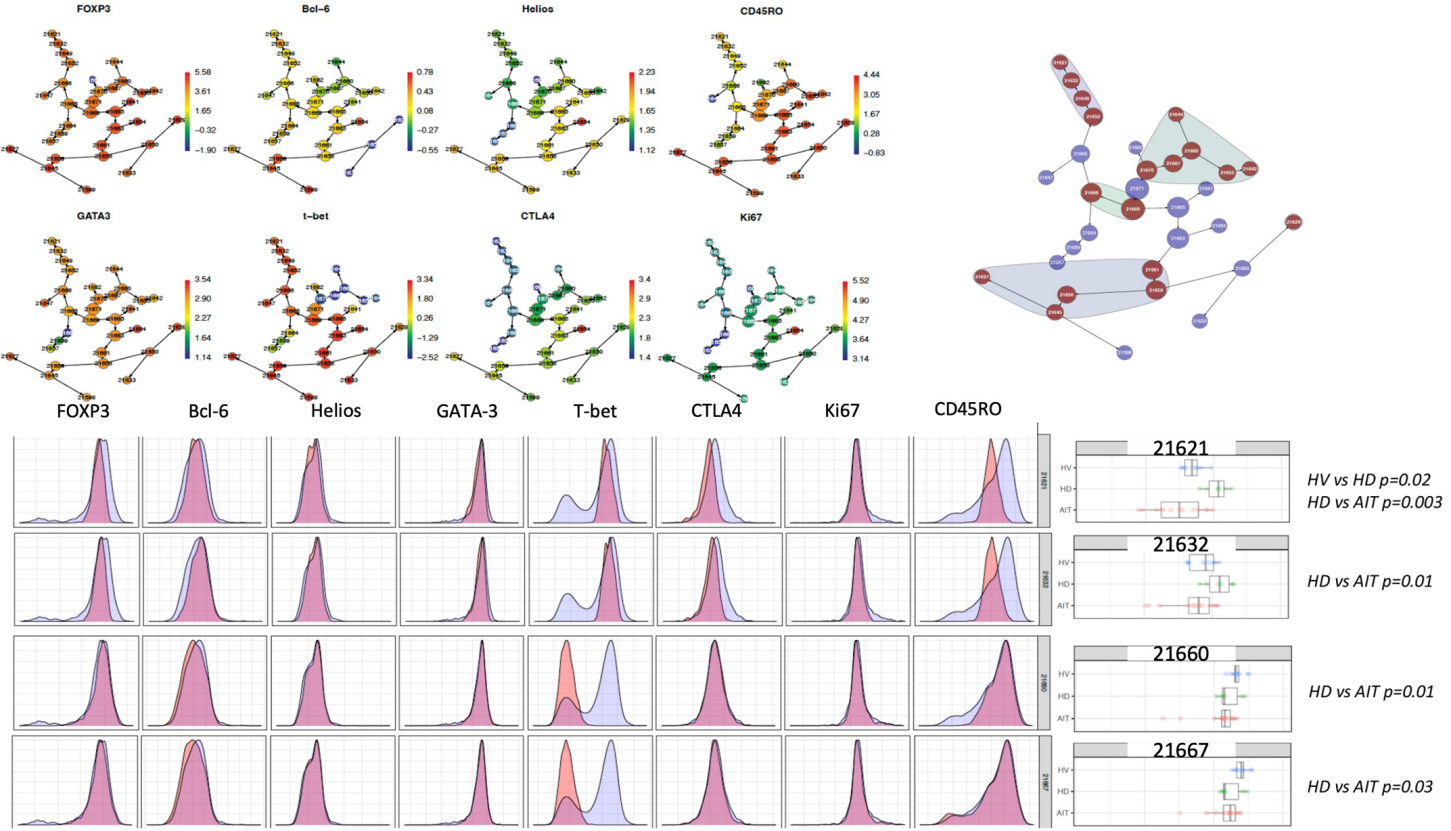
CITRUS analysis of intracellular panel.

Where more than two groups were analysed together, the non-parametric one-way ANOVA test or Kruskal–Wallis test was employed. The *p*-values generated through these calculations were recorded. The CITRUS analysis was carried out using an equal sampling method, with a minimum cluster size of 5, five cross-validation folds, one false discovery rate and 315 events per file for Figure 5 and 714 events for Figure 7.

## Results

We recruited 15 HS individuals on haemodialysis, nine non-sensitised patients on haemodialysis (HD) and compared them to nine HV leukocyte cones samples.

### Patients’ clinical characteristics

The patients’ clinical characteristics are presented in Table 1. In the HD cohort, seven of nine (78%) patients were male and their mean age was 52 ± 9 years. Of them, two (22%) had a background of previous immunotherapy. All were non-sensitised with a cRF of 0 and a mean C-reactive protein (CRP) at the time of blood draw of 9 ± 13.5 mg/L. The mean time on haemodialysis was 2.4 ± 1.4 years. The clinical characteristics of the HS patients are presented in Table 2. The majority were male (9/15, 60%), with a mean age of 49 ± 16 years. Most were from a Black ethnicity (9/15, 60%) and had been on haemodialysis for 5.8 ± 3.3 years. Their mean cRF was 89% and CRP was 4.4 ± 3.7 mg/L. Ten of them had been primarily sensitised through a previous transplant, three through pregnancy and two after transfusion. The transfusion history of eight previously transplanted or pregnant patients was unknown. A comparison of the two groups is presented in Table 3. Patients in the HS group had been on haemodialysis for longer (*p* = 0.005) and more had a background of previous transplantation (*p* = 0.002).

**Table 1:** HD clinical characteristics.

Study number	Gender	Age	Ethnicity	ESRD/comorbidity	Time on HD (years)	Transplant	Pregnancy	Transfusion	Current/Previous IS	cRF	CRP
SIKP 46	F	51	Black—Black British	HTN	2.3	No	No	No	None	0	3
SKIP112	M	41	White—Others	IgA	1.2	No	NA	No	Ustekinumab anti-IL12/IL23	0	1
SKIP113	M	55	Black—any other	Childhood FSGS	4.4	No	NA	No	Previous^a^	0	2
SKIP115	F	52	White—British	Fibrillary GN	1.3	No	No	No	None	0	3
SKIP118	M	52	Black—any other	Secondary FSGS	1.5	No	NA	No	None	0	1
SKIP120	M	57	Other—Any ethnic Gp	HTN Diabetes	2.7	No	NA	No	None	0	41
SIKP 121	M	60	White—British	ADPKD/BG of prostate cancer treated and cleared 2017/currently under investigation for blood in stool	4.7	No	NA	Yes	None	0	20
SIKP 122	M	34	Other-Chinese	Chronic vasculopathy and TIF 50%	0.9	No	NA	No	None	0	2
SIKP 123	M	64	Black-Nigerian	HTN?	2.8	No	NA	No	None	0	5

ESRD, end-stage renal disease; IS, immunosuppression; cRF, calculating reaction frequency; CRP, C-reactive protein; IgA, IgA nephropathy; FSGS, focal and segmental glomerulosclerosis; GN, glomerulopathy; HTN, hypertension; ADPKD, autosomal dominant polycystic kidney disease; BG, background; NA, not applicable; TIF, tubulo-interstitial fibrosis.

^a^ Previous: this patient received in his childhood plasma infusions, steroids, immunosuppression incl. vincristine.

**Table 2:** HS clinical characteristics.

Sample ID	Gender	Age	Ethnicity	ESRD/Comorbidities	Time on HD (years)	Transplant (#)	Transplantectomy	Pregnancy	Transfusion	Current IS treatment	cRF	CRP
AIT 12	M	46	Black—Nigerian	AA Amyloidosis II to yaws	3	Transplant (2)	No	NA		None	100	1
AIT 13	M	52	Black—other African	HTN	9.8	Transplant (2)	Yes	NA		None	100	11
AIT 47	M	51	Black—Nigerian	Unknown	8.4	Transplant (1)	Yes	NA		None	100	2
AIT 61	F	35	White—any other	HTN/atrophic right kidney	6.3	—	—	Yes	Yes		100	3
AIT 63	M	36	Black—Caribbean	FSGS/HTN	2.8	Transplant (1)	No	NA		Pred 5mg	98	12
AIT 69	F	22	Black—Caribbean	Renal dysplasia/Heterozygous HNF1beta mutation/NODAT	4.4	Transplant (1)	No	No		Pred 2.5 mg/Advagraf® 4 mg	100	1
AIT 70	M	24	Asian—any other	Nephronophthisis	6	Transplant (2)	Yes	NA		None	99	1
AIT 72	F	68	Other—any ethnicity	Diabetes II/HTN	1.4	—	—	Yes	Yes		99	1
AIT 73	M	66	Asian—any other	HTN	1.3	—	—	NA	Yes		23	3
AIT 74	F	67	Black—Caribbean	Diabetes II/HTN	11.4	—	—	Yes	Yes		77	7
AIT 75	M	59	Black—any other	DTII ++/HTN/Chemo for relapses DLBCL 2012	4.2	—	—	NA	Yes		56	7
AIT 76	F	50	White—any other	Unknown	9.4	Transplant (2)	No	No	Yes	None	100	1
AIT 77	M	48	Black—other African	HTN	2.9	Transplant (1)	Yes	NA		None	100	6
AIT 78	M	76	Black—any other	HTN	8.3	Transplant (1)	No	NA		Pred 1mg	77	3
AIT 79	F	29	White—British	Congenital NS/BL nephrectomies	7.5	Transplant (1)	No	No	Yes	Pred 10 mg/Adoport®2mg	99	6

ID, identification; ESRD, end-stage renal disease; HD, haemodialysis, #, number; IS, immunosuppression; cRF, calculated reactive frequency; CRP, C-reactive protein; M, male; F, female; HTN, hypertension; FSGS, focal and segmental glomerulosclerosis; NODAT, new-onset diabetes after transplantation; Chemo, chemotherapy; DLBCL, diffuse large B cell lymphoma; NS, nephrotic syndrome; BL, bilateral; NA, not available; Pred, prednisolone; mg, milligram.

**Table 3:** Clinical characteristics of SIKP and HS patients.

	SIKP	HS	*p*
Gender (M/F)	7/2	9/6	0.65
Age	52 ± 9	49 ± 16	0.57
Ethnicity			
Black	4	10	0.4
White	3	3	0.63
Other	2	2	0.61
ESRD			
HTN	4	5	0.67
Diabetes	0	2	0.51
Glomerulopathies	4	2	0.15
Other	1	6	0.19
Time on haemodialysis (years)	2.4 ± 1.4	5.8 ± 3.3	**0**.**005**
Background of IS apart from Transplant	2	1	0.53
Currently on IS	1	4	0.61
Background of Transplant	0	10	**0**.**002**
CRP (mg/L)	9 ± 13.5	4.4 ± 3.7	0.9

ESRD, cause of end-stage renal disease; HTN, hypertension; IS, immunosuppression; cRF, calculated reactive frequency; CRP, C-reactive protein.

Values in bold are considered statistically different.

### Human blood T follicular regulatory cells are CXCR5+CCR7+CD45ra+ICOS-PD-1-

**Figure 1:**
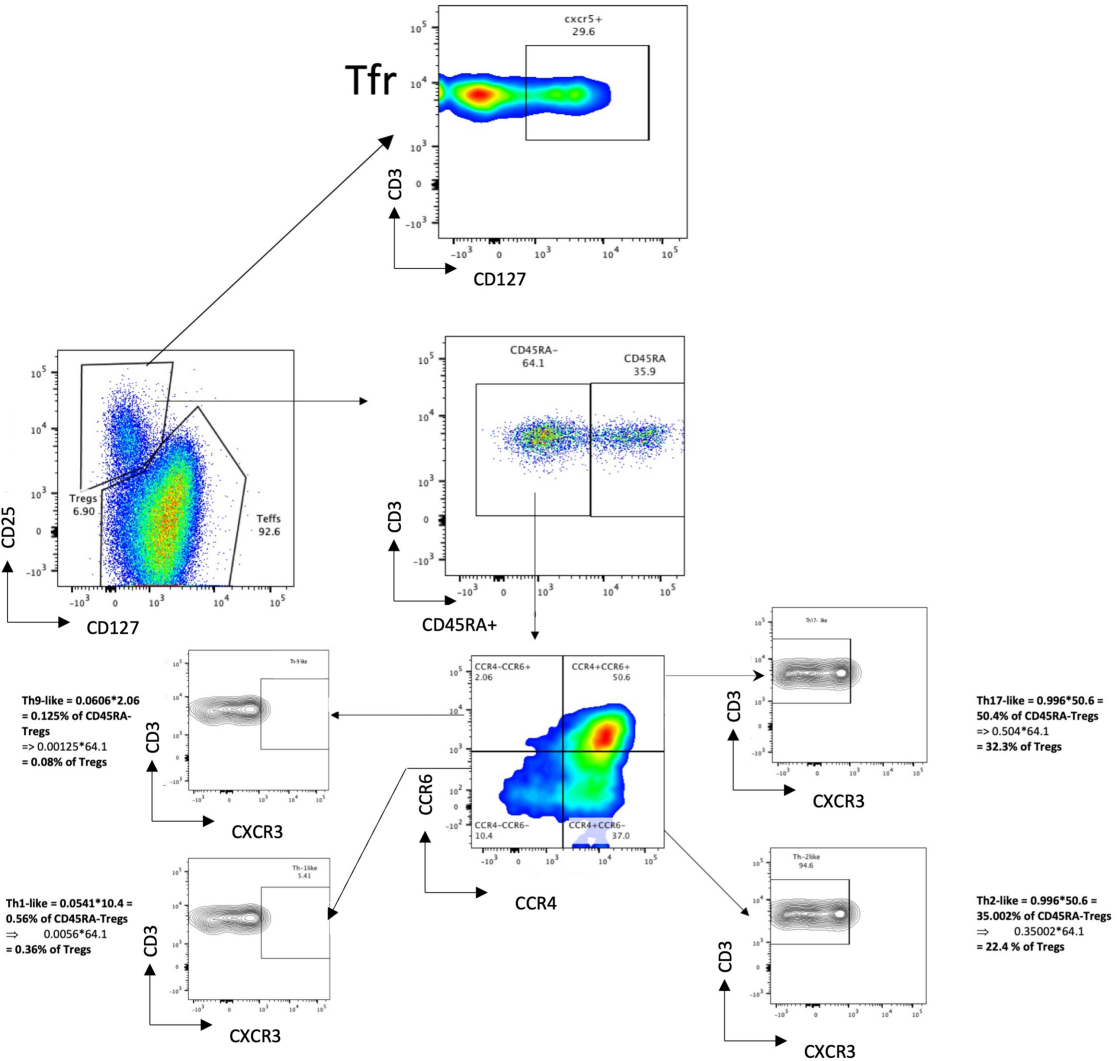
Gating strategy to identify Th-like subpopulations of Tregs.

**Figure 2:**
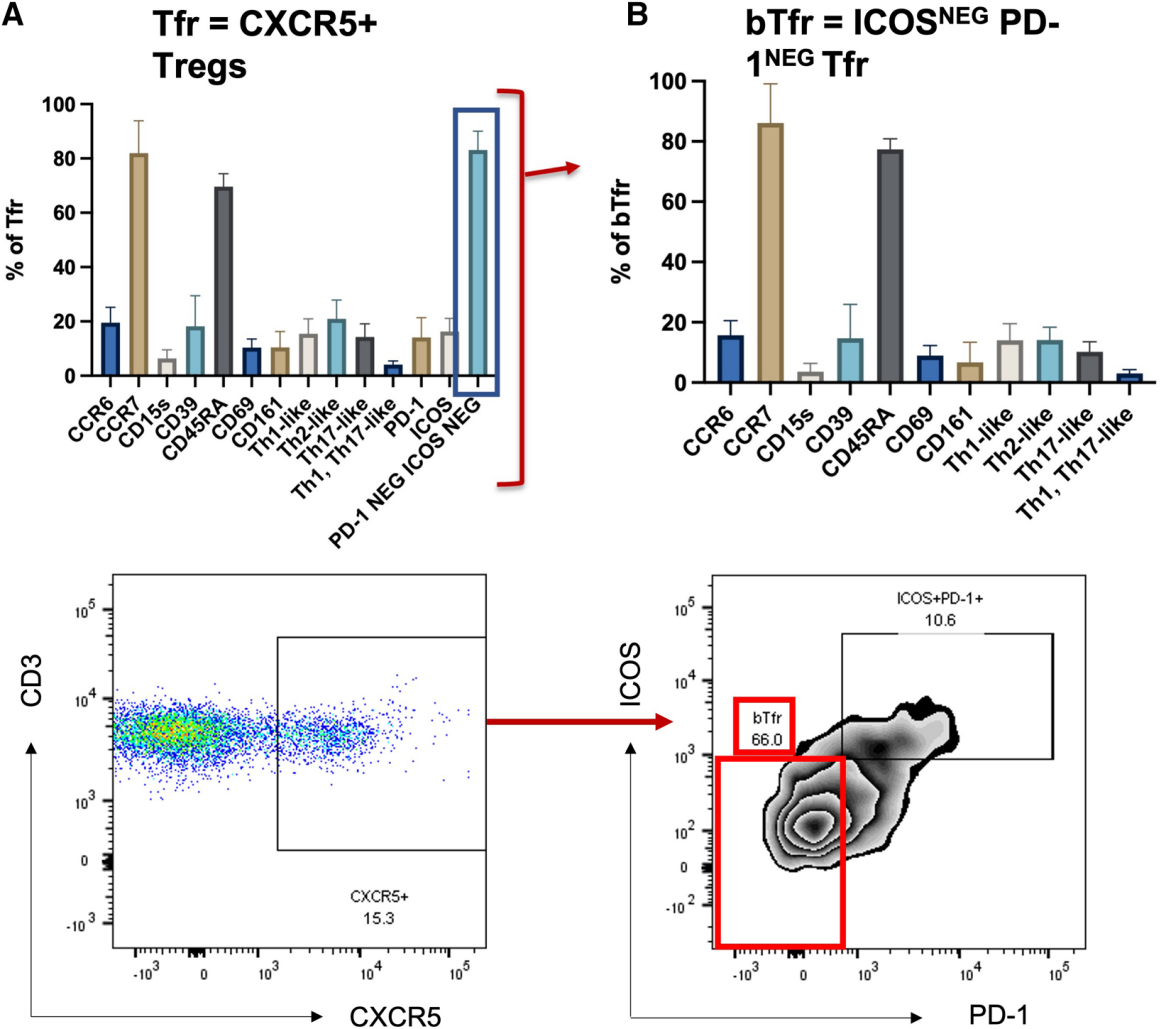
Tfr and bTfr extracellular phenotyping. Description of expression of different markers in the Tfr population (CXCR5+ Tregs) (left panel, **A**) and on the PD-1^neg^ and ICOS^neg^ (bTfr) subpopulation (right panel, **B**). The bTf is obtained by selecting PD-1 negative and ICOS negative cells out of the Tfr population.

The Treg phenotype was studied in nine samples derived from the leucocyte cones of healthy volunteers (the gating strategy is described in Figure 1). The phenotype of blood Tfr (circulating T follicular regulatory cells, called bTfr) has mainly been described in mouse models (3); therefore, we started by identifying the markers expressed by the Tfr population in the blood of healthy individuals. Tfr were defined as Tregs that are CXCR5+ and the markers expressed by them are shown in Figure 2A. The majority of bTfr (defined as ICOS and PD-1-negative CXCR5+ Tregs) looked similar to Tfr and were CCR7^+^ (able to home to secondary lymphoid organs) and CD45RA^+^ (naïve) (Figure 2B), supporting the hypothesis that bTfr come from a “truncated” germinal centre response (3). bTfr could be human Tfr that have gone into the T cell zone within the lymph node, have been activated by the dendritic cell, but probably not enough to go through the germinal centre reaction and therefore recirculate into the blood (Supplementary Figure S1).

### Highly sensitised patients have more CD161^+^ Tregs compared to HV and unsensitised HD

**Figure 3:**
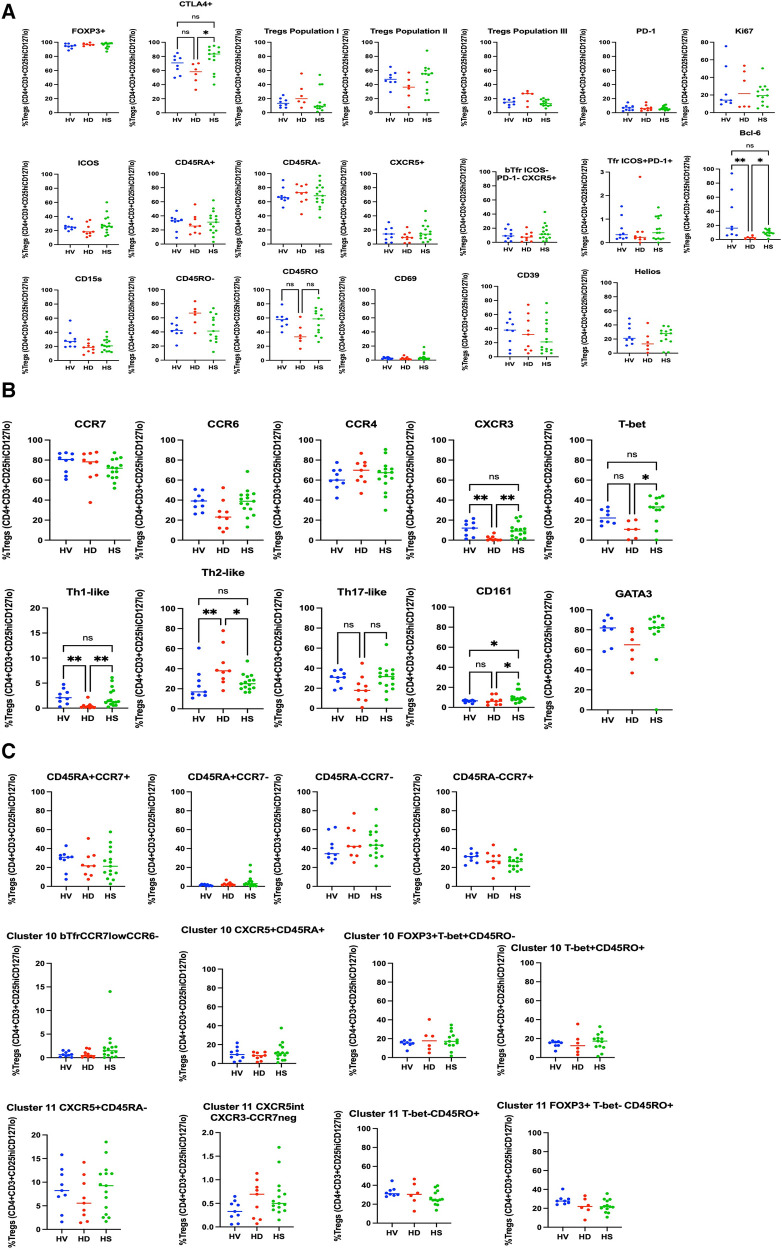
Phenotyping analysis of Tregs in HV, non-sensitised HD, and HS patients. In (**A**) some common Tregs markers, and the population I, II and III are presented. bTfr were defined as CXCR5^+^PD-1^−^ICOS-Tregs. In (**B**) the markers associated with the Th-like populations are presented. There was no difference in the CD45RA/CCR7 groups (**C**), but HS patients had a trend towards increase in the CD45RA^+^CCR7^−^ naïve Tregs population (*p* = 0.0817).

**Figure 4:**
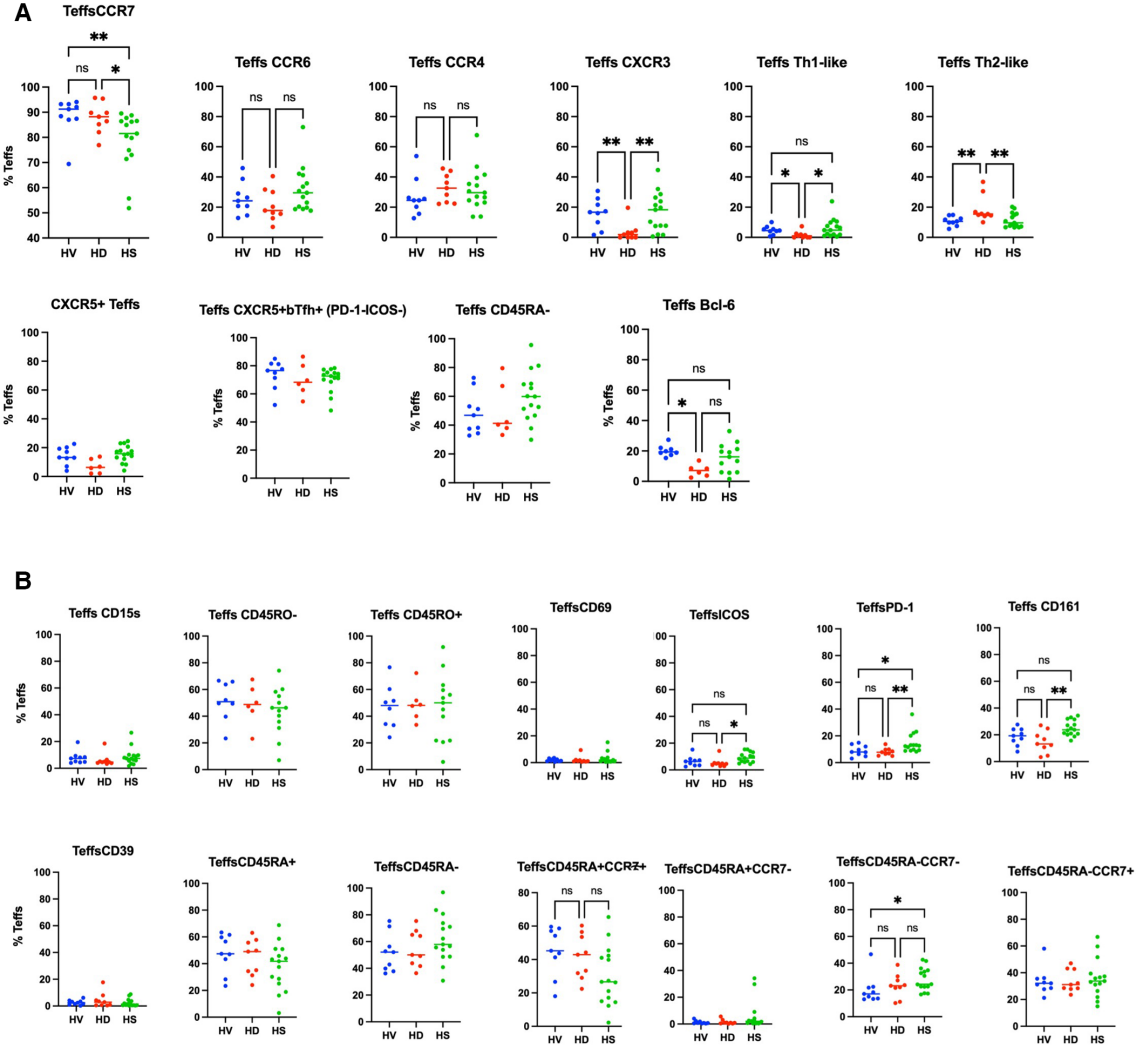
Phenotyping analysis of Teffs in HV, non-sensitised HD, and HS patients. In (**A**) some common Teffs markers are presented. In (**B**) the markers associated with the Th-like populations and subpopulations based on the expression of CD45RA and CCR7 are presented. There was no difference in the CD45RA/CCR7 groups (**C**), but HS patients had a trend towards increase in the CD45RA^+^CCR7^−^ naïve Tregs population (*p* = 0.0817).

We compared HS Treg phenotyping to unsensitised HD patients and healthy volunteers. The analysis was extended to patients who had been sensitised through transfusion and they had a different Treg signature (see Supplementary Material and Figures S2–S4). There was no difference in the proportion of Tregs between the three groups (Supplementary Figure S5). In a biased flow cytometry analysis, unsensitised HD patients had a reduction in Th1-like Tregs (*p* = 0.0026) and Teffs (*p* = 0.0085) and an increase in Th2-like Tregs (*p* = 0.0076) and Teffs (*p* = 0.005), due to a decrease in CXCR3 expression (*p* = 0.0030 and *p* = 0.0079, respectively) (Figure 3A for Tregs and Figure 4A for Teffs). They trended towards a decreased expression of CCR6 (*p* = 0.0637) and CD15s (*p* = 0.0644) in Tregs and displayed a trend towards a decrease in the Tfh population (CXCR5^+^ Teffs), even if non-significant (*p* = 0.0710).

HS patients had a higher proportion of CD161+ Tregs compared to HV and non-sensitised HD patients (Figure 3A) (*p* = 0.02). They had a smaller proportion of CCR7^+^ Tregs compared to HV and HD, and a smaller proportion of Tregs expressing ICOS (*p* = 0.0138) and PD-1 (*p* = 0.0112) (Figure 3B). They had a tendency towards an increase in the CD45RA^+^CCR7^−^-naïve Tregs population (*p* = 0.0817). They had a decrease of CCR7^+^ Teffs (*p* = 0.009), a lower proportion of CD45RA^−^CCR7^−^ Teffs compared to HV (*p* = 0.0409), balanced by a trend to an increase of the CD45RA^+^CCR7^+^ Teffs population (*p* = 0.0553). There was no difference in the expression of the other surface markers, the numbers of bTfr, of ICOS^+^PD-1^+^Tfr and the T-bet^+^CXCR5^+^CD45RO^−^ICOS^−^PD-1^−^ Treg cluster- and T-bet^−^CXCR5^int^CD45RO^+^ICOS^−^PD-1^int^ cluster, as also described by Louis et al. (4).

### HS patients have more of a Th17-like CD161^+^ Treg cluster (CCR6^+^CCR4^+^CXCR3^−^ CD39^+^CD15s^+^ICOS^−^CCR7^−^CD161^+^)

**Figure 6:**
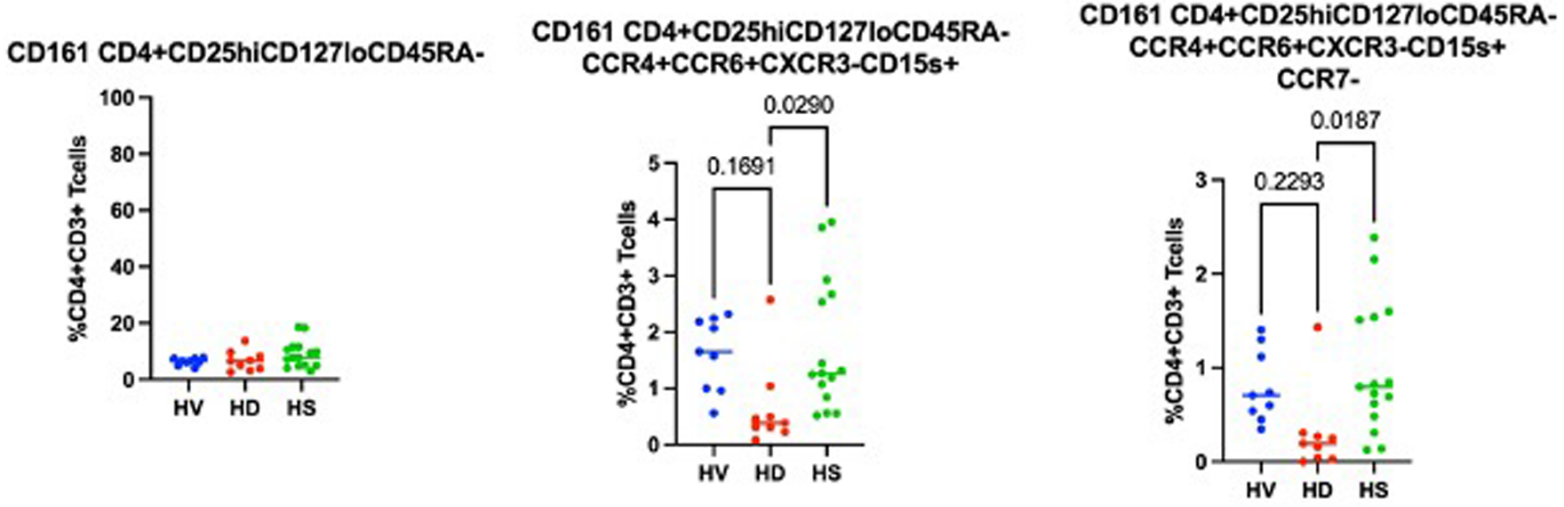
Biased analysis of cluster iii.

Using a CITRUS analysis (available on Cytobank), we compared Tregs from HD, HV and HS patients. HV and HS patients had the same proportion of CD69^−^ clusters (Figure 5) (i) and a Th17-like CD161^+^ Treg cluster (iii.) compared to unsensitised HD. Unsensitised HD had higher proportion of three different CD69^−^ Tregs clusters (i.). Both unsensitised HD and HS patients had a defect in an ICOS^+^Th17-like effector Tregs cluster (ii. 9752, ICOS^+^ CCR7^−^ CCR6^+^CCR4^+^CXCR3^−^CD15s^++^CD39^+^) compared to HV (Figure 5). HS patients had a trend towards more of a Th17-like CD161^+^ Treg cluster (cluster iii., CCR6^+^CCR4^+^CXCR3^−^ CD39^+^CD15s^+^ICOS^−^CCR7-CD161^+^). Interestingly, by using a biased analysis, we were able to identify that HS patients had more of cluster iii compared to unsensitised HD patients (Figure 6). This cluster of Tregs was particularly interesting, as it has been identified as a Th17-like CD161^+^ Tregs cluster in patients with chronic arthritis (13).

### Non-sensitised HD patients had more of a T-bet^+^CD45RO^−^ Tregs population and HS patients had a lower proportion of two T-bet- CD45RO^+^ Tregs clusters compared to HD patients

In a high-dimensional analysis using CITRUS as a clustering algorithm and focusing on intracellular markers, we found that unsensitised patients on dialysis had a trend towards more of a FOXP3_lo_ T-bet^+^CD45RO^−^ population (cluster 21621 and 21632) compared to HV and to HS patients (Figure 7). HS patients had a lower proportion of two T-bet^−^ CD45RO^+^ Treg clusters (21660 and 21667) compared to non-sensitised HD patients (*p* = 0.01 and *p* = 0.03, respectively). These two groups of clusters were of particular interest as they look very close to the ones identified by Louis et al. (4), which were associated with the presence of DSA only (T-bet^+^ CD45RO^−^ Tregs) and DSA/ABMR (T-bet^−^ CD45RO^+^ Tregs).

## Discussion

This is the first study presenting a deep Treg phenotype in HS patients. We demonstrated that they had more of a Th17-like CD161^+^ effector Tregs from population III (CD4^+^CD25^hi^CD127_lo_CD45RA^−^) (cluster iii) compared to unsensitised HD patients.

We described for the first time the presence of Tfr in the blood (called bTfr) in non-sensitised HD and AIT patients and this study is one of the first presenting the presence of CXCR5^+^ Tregs circulating Tregs in HV (4). The identification of bTfr has come mainly from mouse models (8–11) and there are only a few studies that looked at bTfr specifically in HV (3). We confirmed that bTfr are ICOS^−^PD-1^−^, and express a high level of CCR7 and a high level of CD45RA. This supports the hypothesis of the “truncated germinal centre response,” where bTfr could be naïve Tfr that may have interacted with the DC within the T cell zone, but without being activated and would circulate back in the blood [see (3) and Figure 2].

The different groups of patients described have some common characteristics. The HS and unsensitised HD groups are not different for most of the controllable characteristics: age, sex, ethnicity, inflammatory status and cause of end-stage renal disease (ESRD). There was a tendency towards the male sex in the unsensitised HD group (7/9) as female patients are more likely to be sensitised through pregnancies on the top of the risk of being sensitised through blood transfusions. They were different in the cRF, but this was one of the inclusion/exclusion criteria; therefore, some differences were to be expected. In addition, there was a difference in the length of time on dialysis between the two groups. Patients in the SIKP group were on dialysis for a shorter period (2.4 ± 1.4 years) compared to patients in the HS group (5.8 ± 3.3 years) (*p = *0.005). This introduces a bias in the way we can interpret the phenotyping data; however, there was no way to mitigate this bias. Patients with low cRF are much easier to transplant and therefore do not stay on dialysis; therefore, it was more difficult to find patients on the waiting list who had been there as long as the HS patients.

The type of sensitisation and outcomes after transplantation have not been described extensively. One review by Scornik et al. (12) discussed the impact of different routes of sensitisation and the risk of immunisation. They clearly stratified the immunogenicity from low to high immunogenicity as follow: Recent transfusion < Pregnancy alone < Multiple transfusion = Pregnancy + transfusion = Transplantation < Transplantation + Transfusion. In our study, patients who had been sensitised through transfusion had a higher number of ICOS^+^ Tregs (*p* = 0.049), CCR4^+^ Tregs (*p* = 0.0452), a lower proportion of CCR7^+^ Tregs (*p* = 0.0413), and a trend towards more population II Tregs (*p* = 0.0574) and Th17-like Tregs (*p* = 0.0631). This means that patients who had been sensitised through blood transfusion only (AIT 73 and AIT 75) had more Th17-like effector Tregs able to home towards the lymphoid organs. This may relate to the fact that patients who have been immunised by blood transfusion have less immunogenicity. These results need to be validated in a bigger cohort.

In the unbiased phenotyping analysis, our initial hypothesis was that non-sensitised patients on HD would have a different phenotype compared to HV, and that HS patients would have another signature phenotype compared to both HV and non-sensitised HD patients. However, first, we demonstrated that non-sensitised HD had fewer Bcl6^+^ Tregs and Teffs, and fewer Th1-like Tregs and Teffs, compared to healthy volunteers. We did not find any difference in the numbers of Tregs as a proportion of CD4^+^ cells. This is different from the previous literature describing either increases in the number of patients on HD (13, 14), or a decrease in the number (15), but is consistent with the work performed by Afzali et al. (16). Afzali et al. compared the Tregs phenotype in non-sensitised HD patients and healthy volunteers. They identified that Tregs from patients on HD had fewer Tregs from population II, that age did not impact the number of Tregs in this population, and there were no differences in expression of CD39, FOXP3, HLADR and CD27 by Tregs in HD and HV.

This study is, to the best of our knowledge, the first to phenotype Tregs in HS patients. When recruiting patients, we recruited them on a minimum immunosuppression to reduce as much as possible the impact of treatment on the T cell populations present. Interestingly, in the biased analysis, HS patients had more CD161 Tregs, fewer CCR7^−^ Teffs, more CD45RA-CCR7^−^ Teffs (memory Teffs capable to home to the germinal centre) and more PD-1 Teffs. Afzali et al. (16) demonstrated that Tregs from patients on haemodialysis had a lower proliferation rate and a decreased suppressive ability, which recovered after culture using IL2 and Rapamycin. While the functional capacities of Tregs were not studied in this manuscript, based on this previous published work, expanding Tregs using Rapamycin could be promising, particularly if it could be confirmed that the expansion process is reducing the number of cells producing IL-17, as demonstrated by Afzali et al. More interestingly, an unbiased analysis using CITRUS (Figure 7) confirmed that HS patients had more of a CD161^+^ effector Tregs from population III (CD4^+^CD25^hi^CD127_lo_CD45RA^−^) (cluster iii) compared to unsensitised HD. This is particularly relevant as our group identified this specific cluster present in the inflamed joints of patients with inflammatory arthritis (17) and it could be a subset of Tregs involved in inflammation associated with sensitisation. Moreover, our group demonstrated that this specific subset of Tregs was highly suppressive (18) and produce cytokines favouring wound healing. HS patients had a lower proportion of two T-bet- CD45RO^+^ Tregs clusters compared to non-sensitised HD patients (*p* = 0.01 and *p* = 0.03, respectively). Recently, one study compared the Tregs phenotype between HV, patients with ESRD but not on HD and transplant patients (KTx group) (13). A major difference between these groups was that patients in the transplant group had functioning transplants and were on immunosuppression. In that study, they demonstrated that KTx patients showed increased frequencies of naïve and effector memory natural Tregs (nTregs), whereas central memory nTregs were reduced. Patients with ESRD had nTregs (CD4^+^CD25^hi^FOXP3^+^) that expressed higher levels of CD127 but lacked CD154 expression upon activation. However, we found no differences in this population in our analyses, demonstrating the complexity of the different groups.

This is the first study presenting a deep Treg phenotype in highly sensitised patients. We confirmed that HS patients had more of a Th17-like CD161^+^ effector Tregs from population III (CD4^+^CD25^hi^CD127_lo_CD45RA^−^) compared to unsensitised HD patients. This is of particular interest for future areas of research, including strategies to restore a less pro-inflammatory phenotype of Tregs.

## Data Availability

The raw data supporting the conclusions of this article will be made available by the authors, without undue reservation.
