# Supplementary material for: Highly sensitised individuals present a distinct Treg signature compared to unsensitised individuals on haemodialysis
Source: Front Transplant. 2023 Oct 30;2:1165320. doi: 10.3389/frtra.2023.1165320 (PMC11235238; doi:10.3389/frtra.2023.1165320)

Supplementary materials

Figure S1 – Early germinal centre reaction and T follicular regulatory cell maturation process (Dudreuilh et al, Frontiers in Immunology 2021)

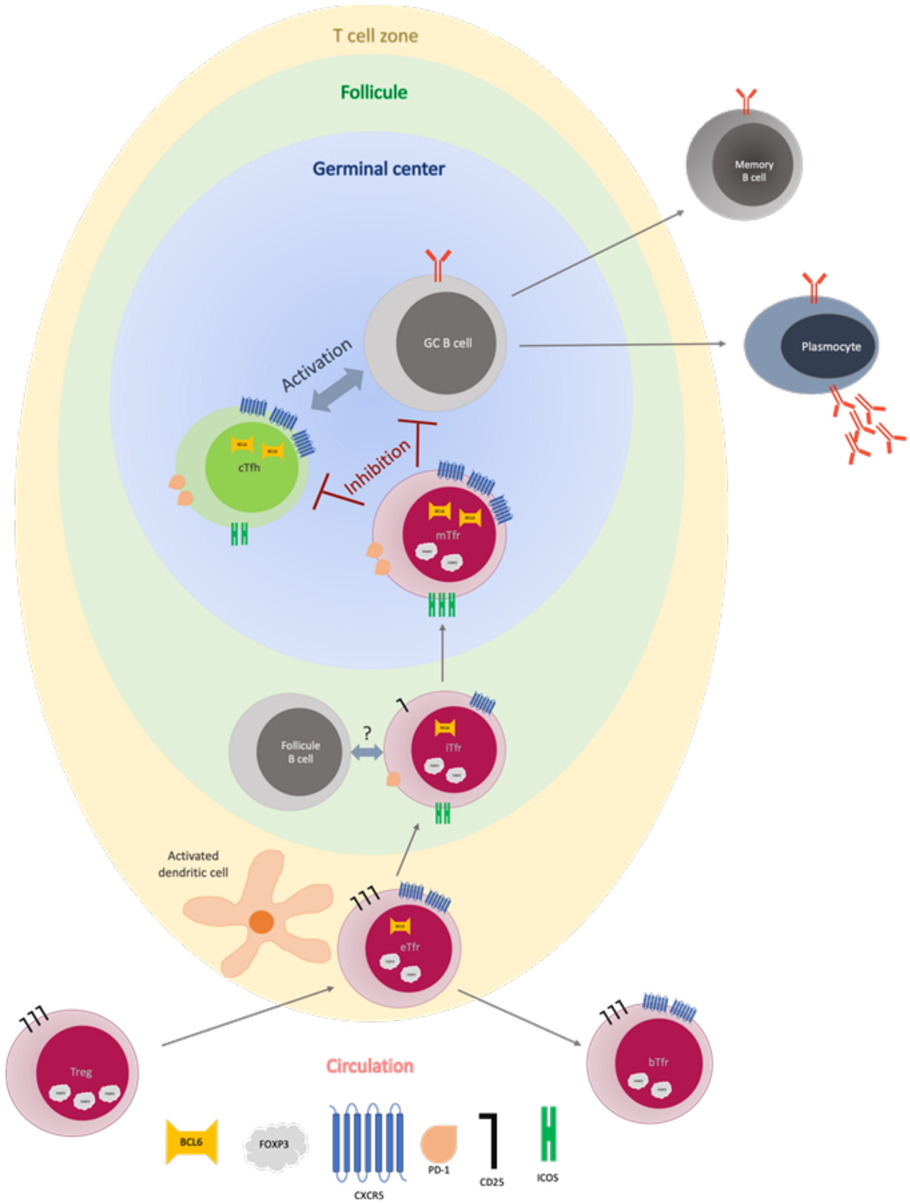

Early Tfr (eTfr) derive from natural regulatory T cells (Tregs) after expression of CXCR5 and Bcl-6 and down regulation of FOXP3. After interaction with activated dendritic cells (DC) in the T cell zone, some eTfr lose expression of Bcl-6 and enter the circulation (blood Tfr-bTfr), while some migrate to the follicle (intermediate Tfr-iTfr), where they interact with the follicular B cells and start expressing PD-1 and ICOS. Eventually, they move to the germinal centre (GC), becoming mature Tfr (mTfr), where they inhibit both the central T follicular helper cell (cTfh) and the GC B cells, leading to regulation of antibody production and B cell differentiation.

Table S1- Table of antibodies for extracellular panel used to phenotype CD4^{+} cells.

Extracellular					
**Fluorochrome**	**Marker**	**Company**	**Clone**	**Ref**	**Quantity**
**BB515/A488 (FITC)**	ICOS	BioLegend	C398.4A	313506	5
**PE**	CXCR5	BioLegend	J252D4	356904	2.5
**PE-CF594**	CD25	BioLegend	BC96	302646	3
**PerCP Cy 5.5**	CCR7	BioLegend	G043H7	353220	7
**PE-Cy5**	CXCR3	BD Bioscience	1C6/CXCR3	551128	3
**PE-Cy7 / PE-Vio 770**	CD161	Miltenyi	191B8	130-113-594	2
**Alexa 647 (APC)**	OPEN				
**Alexa700**	CD3	BioLegend	UCHT1	300424	3
**APC-Cy7**	LD	Thermofisher	Near IR		
**BV421**	CCR6	BioLegend	G034E3	353408	5
**BV510**	CD15s	BD Bioscience	CSLEX1	563529	3
**BV605**	CCR4	BD Bioscience	1G1	562906	5
**BV650**	CD69	BioLegend	FN50	310934	3
**BV711**	PD-1	BioLegend	EH12.2H7	329928	5
**BV786**	CD127	Thermofisher	EBioRDR5	78-1278-42	5
**BUV 395**	CD4	BD Bioscience	SK3	563550	5
**BUV563**	CD45RA	BD Bioscience	HI100	565702	3
**BUV 737**	CD39	BD Bioscience	TU66	564726	1

Quantity in µl.

Table S2- Antibodies for intracellular panel of CD4+ cells

Intracellular					
**Fluorochrome**	**Marker**	**Company**	**Clone**	**Ref**	**Quantity**
**BB515/A488**	Helios	Biolegend	22F6	137214	5
**PE**	FOXP3	TF	PCH101	12-4776-42	7
**PE-CF594**	CD25	BioLegend	BC96	302646	3
**PerCP Cy 5.5**	GATA 3	BD Bioscience	566642	L-50-823	1
**PE-Cy5**	OPEN				
**PE-Cy7**	Bcl-6	BD Bioscience	K112-91	563582	7
**Alexa 647**	t-bet	TF	4B10	48-5825-82	1.5
**Alexa700**	CD3	BioLegend	UCHT1	300424	3
**APC-Cy7**	LD				
**BV421**	CTLA4	BD Bioscience	BNI3	562743	5
**BV 510**	Ki 67	Biolegend	KI67	350518	3
**BV605**	OPEN				
**BV650**	OPEN				
**BV711**	CD45RO	BioLegend	UCHL-1	304236	3
**BV786**	CD127	Thermofisher	EBioRDR5	78-1278-42	5
**BUV 395**	CD4	BD Bioscience	SK3	563550	5
**BUV563**	OPEN				
**BUV 737**	OPEN				

Quantity in µl.

1/ Patients who had been sensitised through transfusion had a different Tregs signature

Within the HS patients' group, we compared the Tregs phenotype of different subgroups (Table S1) using 2-way Anova test, with a Bonferroni correction to account for multiple comparisons. The different subgroups were categorised according to:

1/ the route of sensitisation (Group 1, 2, 3) (see Table S3)

2/ if background of transplantation, patients still on immunosuppression and patients who were not on any immunosuppression (Group 1a, 1b)

3/ if background of transplantation, patients who had a transplant nephrectomy and those who didn't (Group 1c, 1d)

4/ if background of transplantation and nephrectomy, patients who were on IS and the ones who did not (Group 1a, 1e)

Patients who had been sensitised through transfusion (group 3) had a higher number of $ICOS^+$ Tregs (p=0.049) and of population II Tregs ($CD25^{+++}FOXP3^{hi}CD45RA-$ (Miyara)) compared to patients who had been sensitised through transplantation (group 1) (p=0.0574) (Figure 3 & 4). Patients from Group 3 had a higher proportion of Tregs expressing CCR4 (p=0.0452), a lower proportion of Tregs expressing CCR7 (p=0.0413) and a tendency towards a higher Th17-like Treg population (p=0.0631). We compared the Teffs population between all the subgroups and were not able to identify any differences between those groups (see Figure S2-S4).

Table S3- Description of the different subgroups within the HS population, separated by route of sensitisation.

Group 1 Sensitisation by Transplantation	**AIT 12 / AIT 13 / AIT 47 / AIT 63 / AIT 69 / AIT 70 / AIT 76 / AIT 77 / AIT 78 / AIT 79**		
	Group 1a	*Transplantation still on IS*	AIT 63/ AIT 69 / AIT 78 / AIT 79
	Group 1b	*Transplantation not on IS*	AIT 12 / AIT 13 / AIT 47 / AIT 70 / AIT 76 / AIT 77
	Group 1c	*Transplantation with nephrectomy*	AIT 13 / AIT 47 / AIT 76 / AIT 77
	Group 1d	*Transplantation without nephrectomy*	AIT 12 / AIT 63 / AIT 69 / AIT 70 / AIT 78 / AIT 79
	Group 1e	*Tx without Nephrectomy not on IS*	AIT 12 / AIT 13 / AIT 76
	Group 1f	*Tx without Nephrectomy on IS*	AIT 63/ AIT 69 / AIT 78 / AIT 79
Group 2 Sensitisation by Pregnancy	**AIT 61 / AIT 72 / AIT 74**		
Group 3 Sensitisation by Transfusion	**AIT 73 / AIT 75**		

IS, immunosuppression.

Figure S2 – Tregs phenotype of different HS subgroups – part I

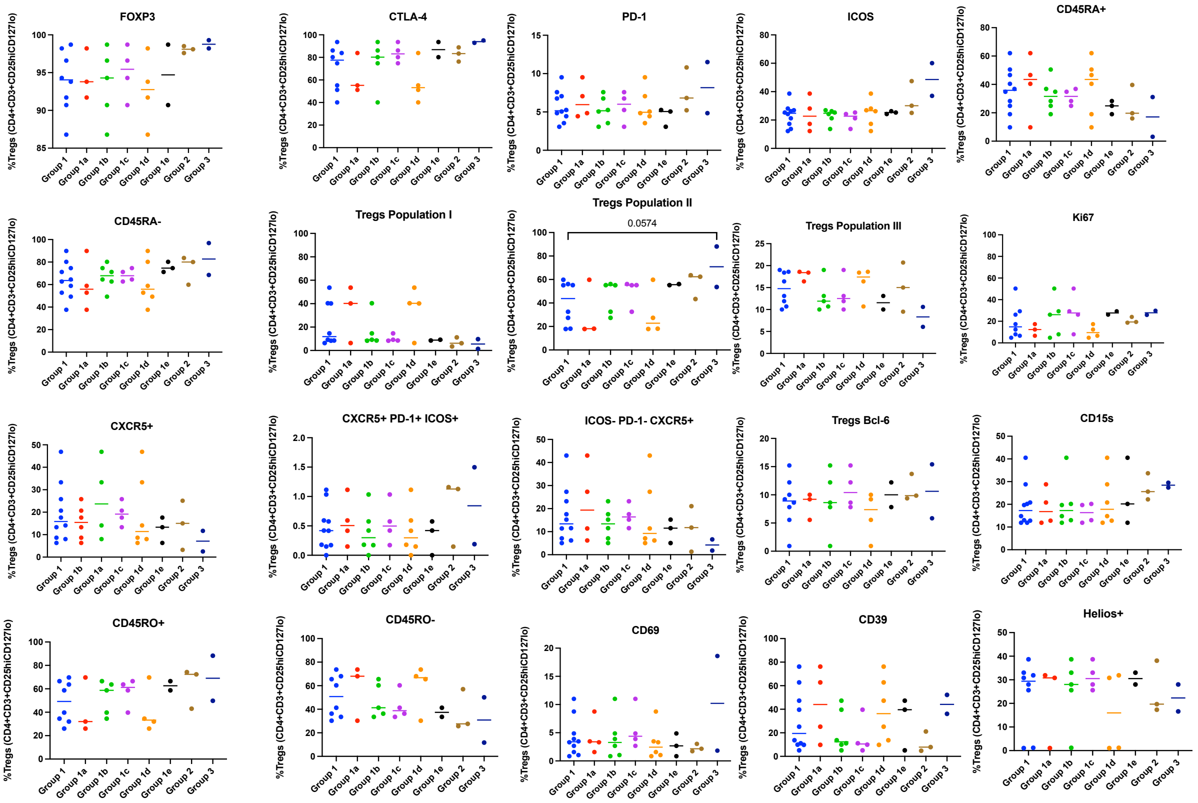

Tregs markers in HS patients depending on their route of sensitisation. Two-ways Anova test were run on the different groups, corrected with Bonferroni to account for multiple comparisons. Only statistically different tests or close to significance are shown.

Figure S3– Tregs phenotype of different HS subgroups – part II

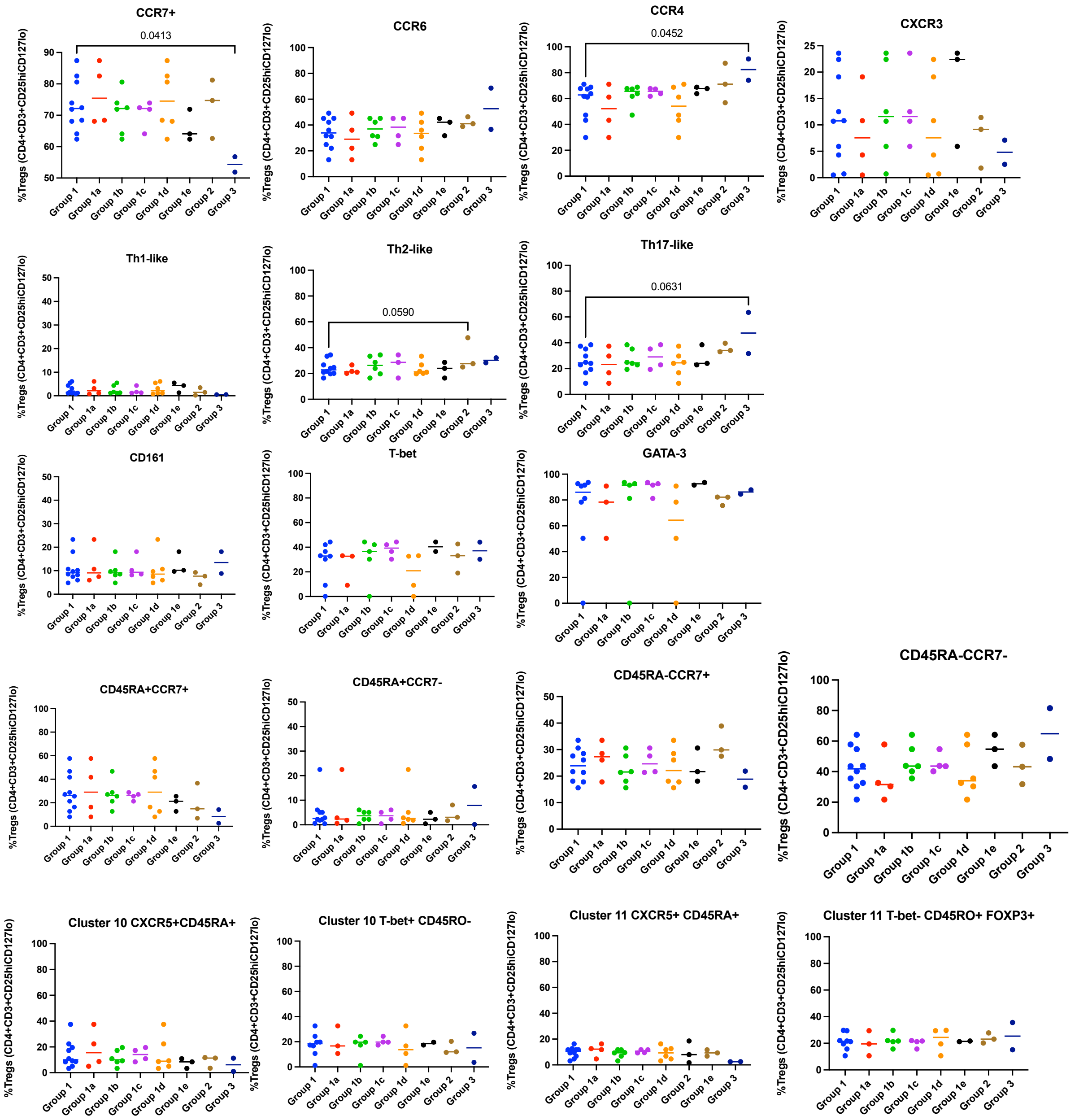

Th1-like, Th2-like and Th17-like populations were gated based on Figure 22 of this manuscript. Cluster 10 and 11 were defined using manual gates in FlowJO® based on the work published by Louis et al (KI 2021).

Figure S4 – T effectors phenotype of different HS subgroups – part II

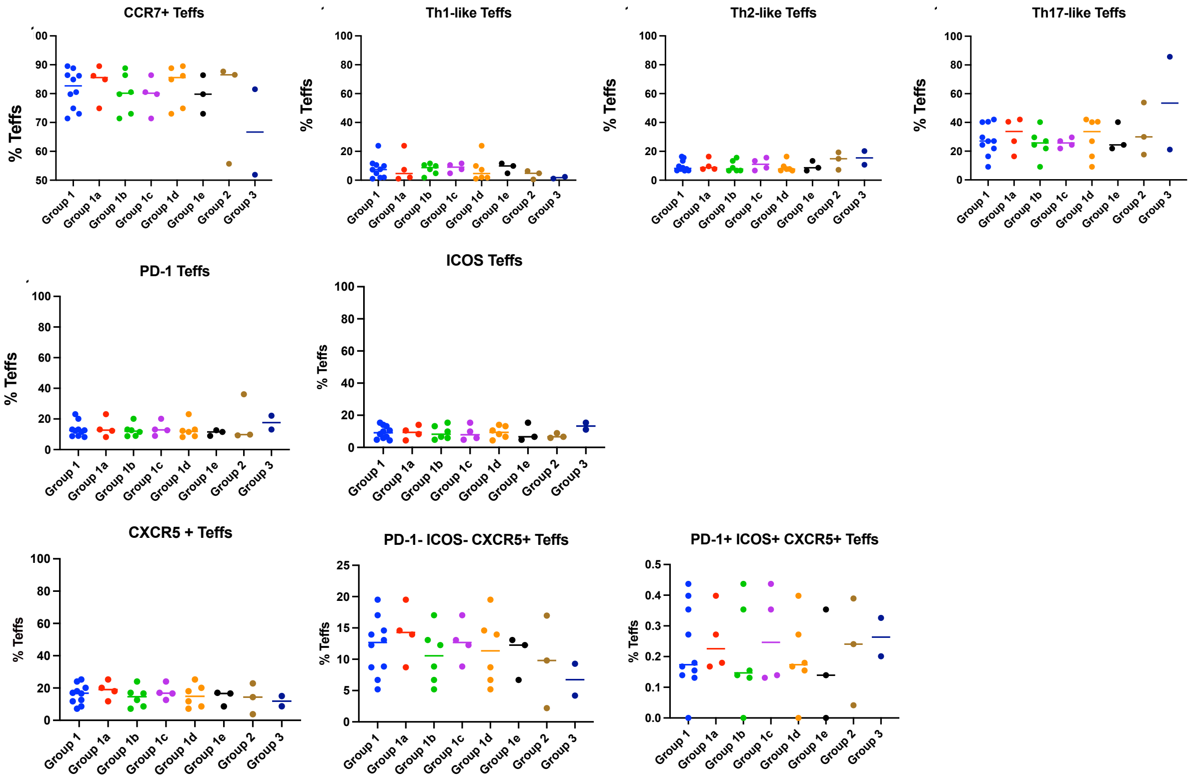

Figure S5- Number of Tregs as percentages of CD4+CD3+ T cells in HV, HD and HS patients.

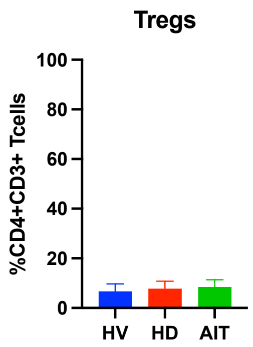

Supplement: Supplementary file 1 [file Datasheet1.pdf]
